# Torsion of Epididymal Cyst: A Case Report With Review of Literature

**DOI:** 10.7759/cureus.51158

**Published:** 2023-12-27

**Authors:** Gurubharath Ilangovan, Manimaran M, Vogu Mounika, Moien AB Khan

**Affiliations:** 1 Radiology, Tagore Medical College and Hospital, Chennai, IND; 2 Radiodiagnosis, Tagore Medical College and Hospital, Chennai, IND; 3 Radiodiagnosis, Tagore Medical College and Hospital, chennai, IND; 4 Family Medicine, College of Medicine and Health Sciences, United Arab Emirates University, Al Ain, ARE; 5 Primary Care, North West London - National Health Service Provider, London, GBR

**Keywords:** india, doppler, acute scrotal pain, ultrasound, cyst, epididymal, torsion

## Abstract

Torsion of an epididymal cyst is one of the rare and least-known causes of acute scrotal pain. Epididymal cysts, when large, can undergo occasional complications like infection or, rarely they might undergo torsion, needing emergency surgery.

We present a case of a 37-year-old gentleman with acute scrotal pain to the scrotum. Testicular torsion was suspected clinically, but sonography revealed a normal-appearing testis and a large left-sided epididymal cyst with internal echoes and dependent debris. A diagnosis of epididymal cyst torsion was suspected based on sonographic findings. Exploratory surgery showed a reddish, inflamed epididymal cyst that had undergone torsion on its pedicle. The cyst was excised leading to symptomatic relief to the patient. Due to the rarity of this condition, such cases are often misdiagnosed clinically as testicular torsion. Ultrasonography helps in aiding the correct diagnosis and the radiologist needs to be familiar with the radiological aspects of diagnosing torsion of epididymal cysts.

## Introduction

Epididymal cysts (EC) are generally benign lesions, with their prevalence varying considerably between children and adults [1]. In the pediatric population, they constitute a relatively rare finding, accounting for approximately 5% to 20% of cases in the available literature, in contrast to their higher incidence among adults [2]. The precise etiology of these cysts is still debated although they have been postulated to stem from congenital anomalies linked to hormonal fluctuations during embryonic development [3]. Typically, ECs are managed conservatively, with spontaneous regression observed in most instances.

However, while epididymal cysts are predominantly asymptomatic, they can undergo torsion upon themselves, culminating in acute testicular pain. While occurrences of such cases are rare, they emphasize the significance of being watchful and conducting a thorough differential diagnosis. This is especially relevant in situations involving children, as the infrequency of EC torsion requires a heightened level of suspicion [4,5]. This case report presents an unusual incidence of acute scrotal pain in a 37-year-old male due to torsion of an epididymal cyst, highlighting the diagnostic challenges and the importance of accurate and timely intervention.

## Case presentation

A 37-year-old gentleman with acute onset of severe scrotal pain was referred to the radiology department with clinical suspicion of testicular torsion. He presented to the emergency department with severe, unremitting pain in the left hemiscrotum which was only mildly relieved by analgesics. He had a history of left-sided scrotal swelling for the past two years for which he had not undergone any investigation. He had no relevant medical, family, or any prior surgical history, and was not on any regular medications. Physical examination revealed a cystic swelling in the left hemiscrotum which was exquisitely tender. The left testis could not be felt separately from the swelling. The patient was afebrile. A working diagnosis of possible left testicular torsion was made, and the patient was referred for an ultrasound examination.

**Figure 1:**
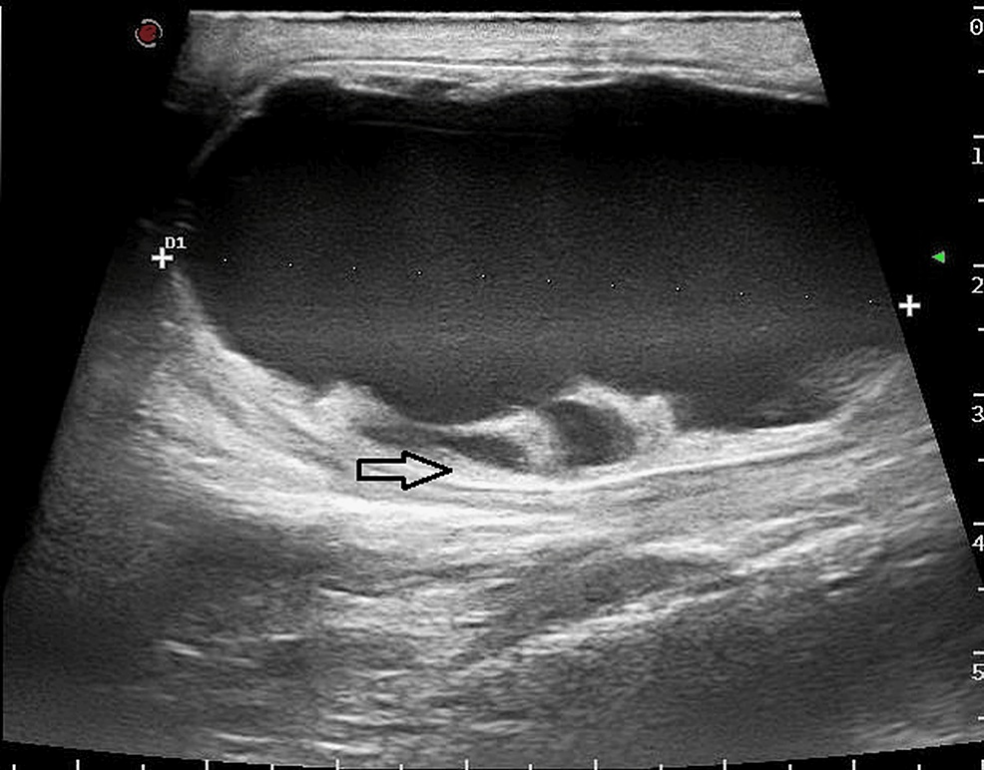
Transverse ultrasound view of the left epididymal cyst with fine internal echoes and thin septae.

**Figure 2:**
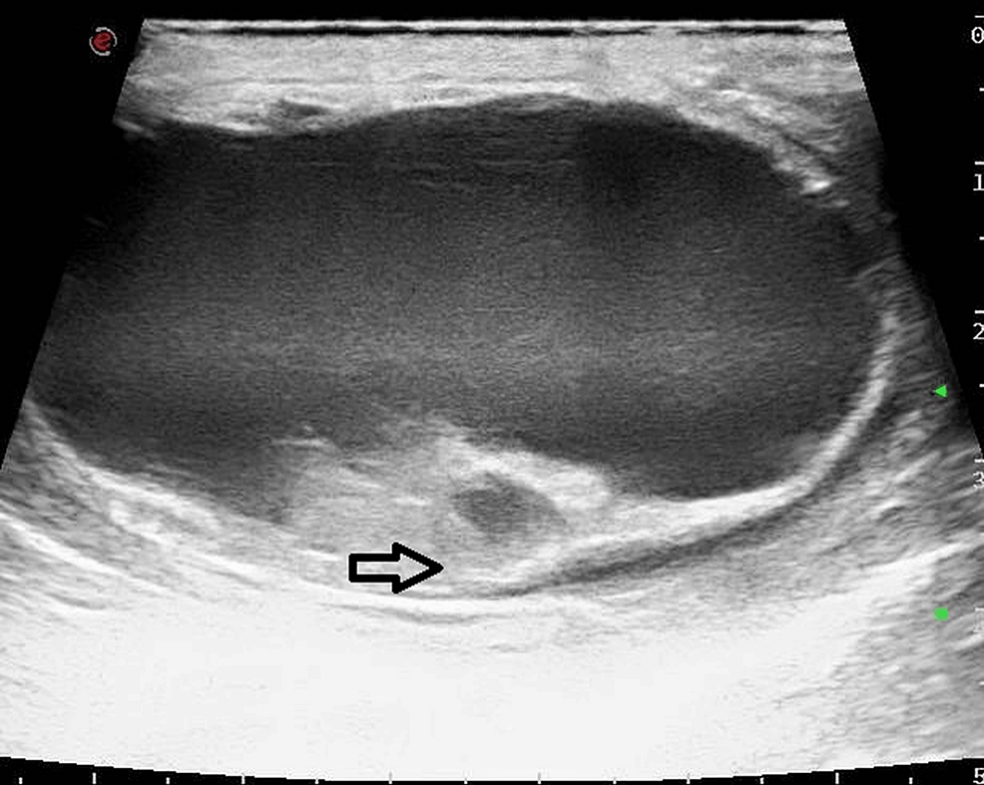
Longitudinal ultrasound view of the left epididymal cyst with fine internal echoes representing blood products.

Sonography revealed a left-sided epididymal fluid-filled cyst of size 5 x 4 x 5 cm arising from the head of the left epididymis (Figure 1), with the left testis revealing no abnormality. The epididymal cyst showed fine free-floating echoes within and a few thin septations (Figure 2). There was no internal vascularity within the cyst. Even though torsion of the cyst was suspected, and a twisted pedicle of the cyst was looked for, clear evidence of a pedicle could not be demonstrated.

**Figure 3:**
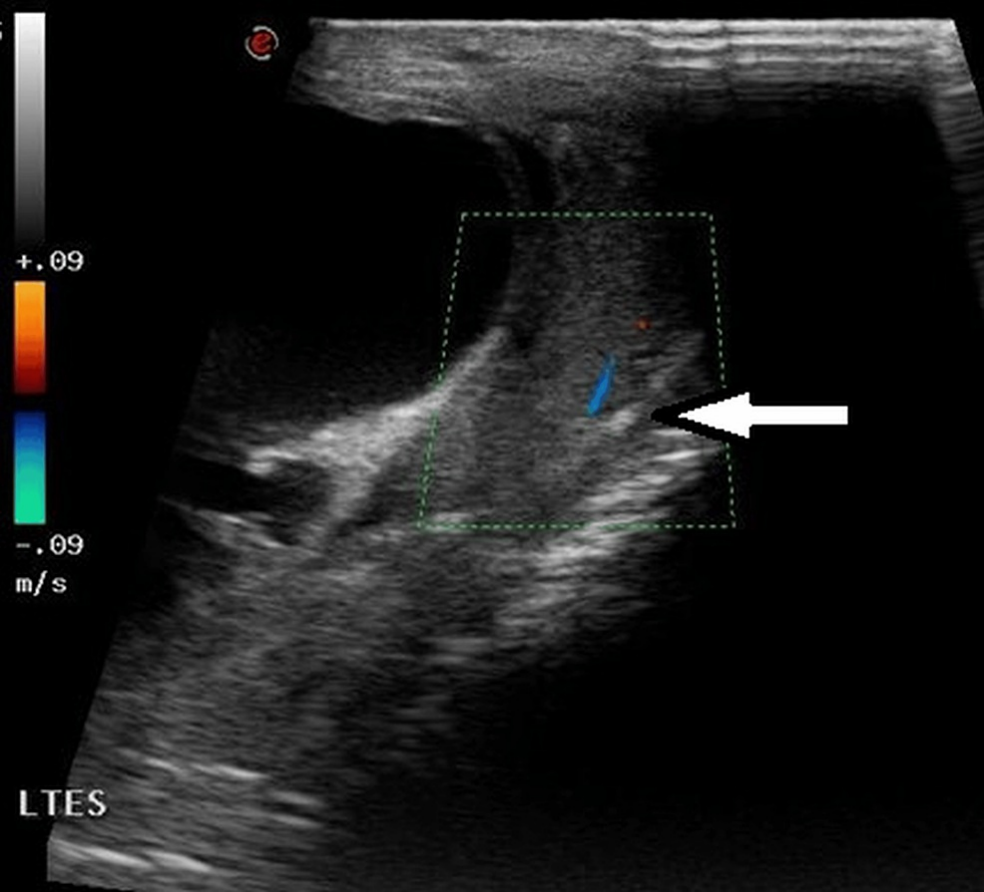
Duplex scan of the left testis demonstrates normal vascularity.

**Figure 4:**
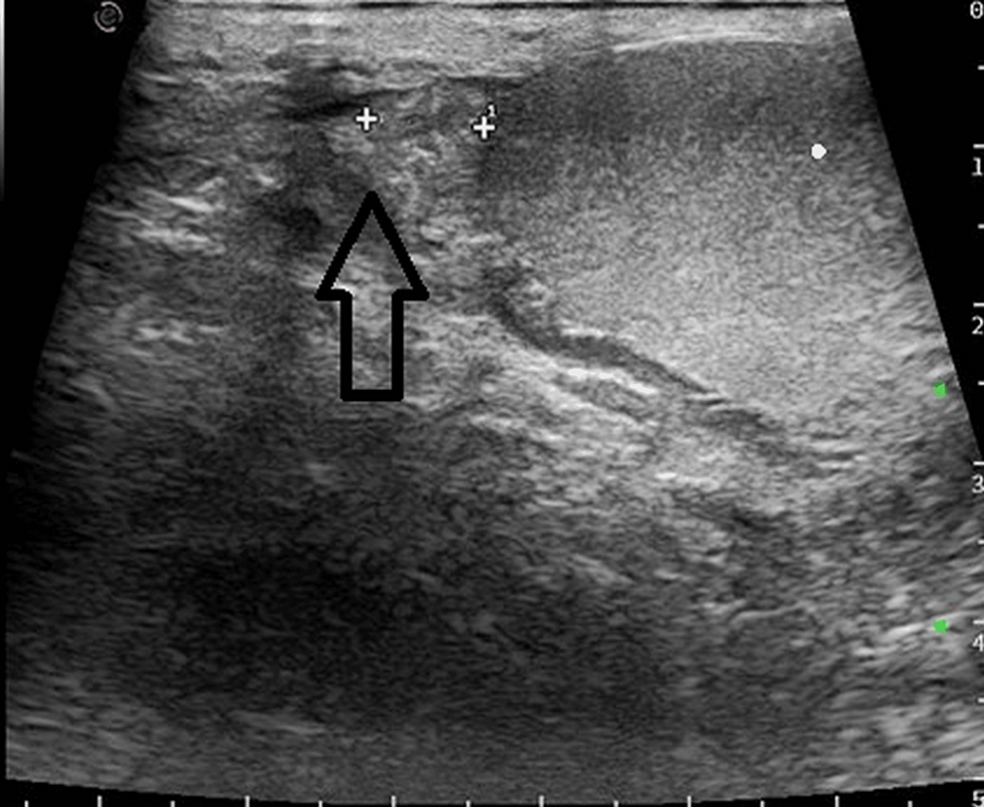
Sonography reveals normal right epididymis.

**Figure 5:**
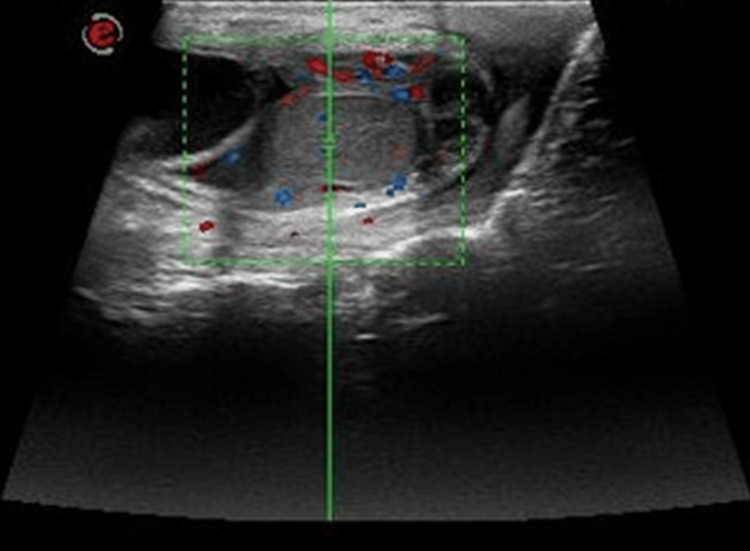
Duplex scan reveals normal vascularity in the right testis.

Both, the testes and right epididymis showed normal vascularity and echogenicity (Figures 3-5).

**Figure 6:**
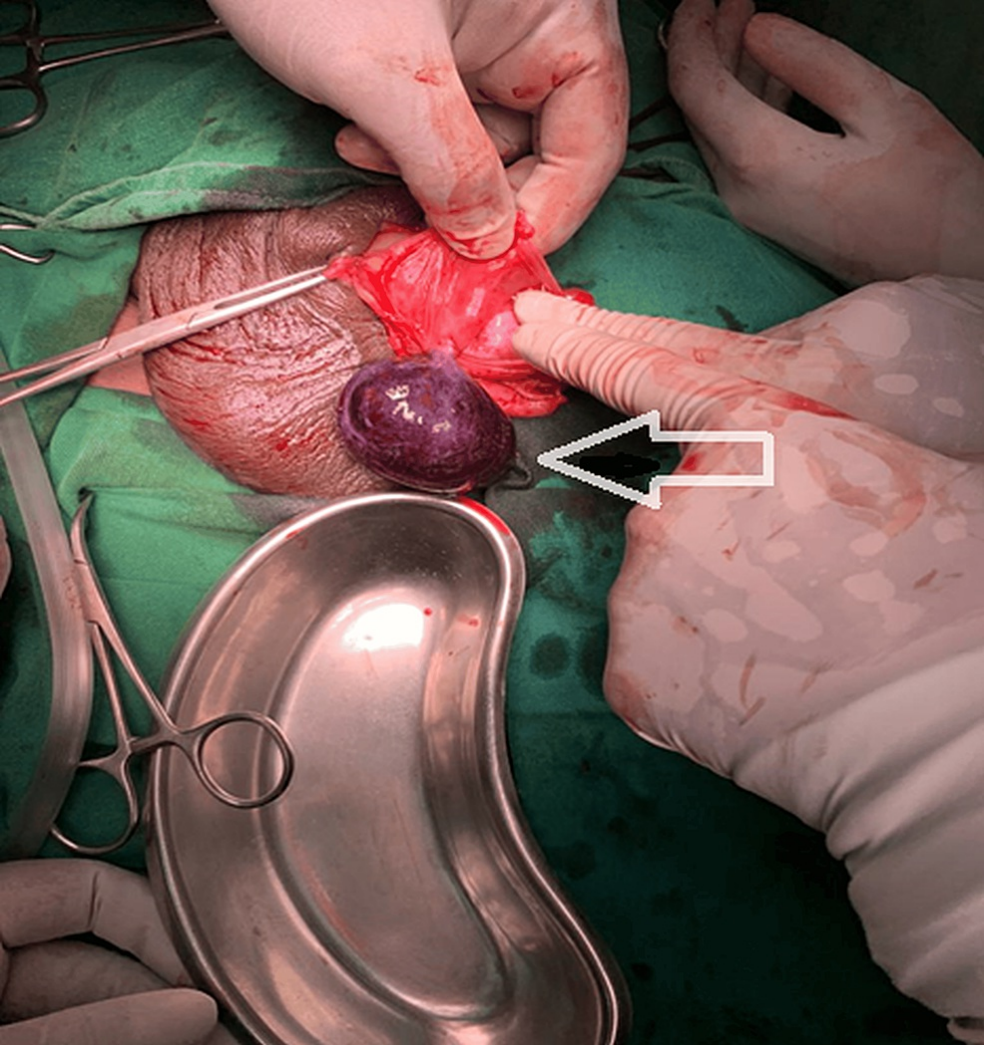
Surgical finding during dissection of the scrotal layers.

Based on the sonographic findings, the possibilities of either torsion of the epididymal cyst or infected epididymal cyst were considered. Infection was seen as a less likely possibility as the patient had no constitutional symptoms and his inflammatory markers were within normal limits. The patient was posted for emergency surgery after adequate explanation and informed consent. The scrotum was dissected down to reach the cyst. The cyst was seen arising from the head of a left epididymis and was twisted over two whole turns (720°) (Figure 6).

The cyst pedicle was cauterized and dissected, and the cyst was removed in its entirety. The cyst appeared reddish and inflamed and was found to contain serosanguinous fluid. Microscopic examination revealed RBCs, pus cells, and a few epithelial cells within the cyst fluid. Post-surgery, the patient experienced near-complete cessation of pain. The patient was observed for three days and then discharged. A follow-up ultrasound a week later showed normal findings, and the patient remained symptom-free. Furthermore, a follow-up examination was normal after 3 months. 

## Discussion

To our knowledge, this is the third case reported from India [6,7] with only around 10 cases reported worldwide [8]. Epididymal cysts are very common and usually asymptomatic benign lesions occurring in almost all ages, predominantly in adults. The incidence in children approaches 20% [2]. They can be palpated on physical examination as smooth, extra-testicular masses [9]. They are usually treated conservatively, but they may be treated surgically when they cause symptoms like pain or when they become very large [9]. Epididymal cysts that are incidentally discovered on ultrasound are usually smaller in size. Larger cysts are less common but have a higher propensity to undergo torsion. It is crucial to recognize that up to 60% of epididymal cysts may spontaneously regress, particularly if they are smaller than 3 cm [4]. Elective surgery is generally advised for cysts larger than 10 mm, symptomatic, or persistent [10]. In cases managed conservatively, patients and guardians should be educated about acute scrotum symptoms and when to seek immediate medical attention.

This case presents various potential causes for acute scrotal conditions, including testicular torsion, epididymorchitis, torsion of the testicular appendix, injury, and strangulated inguino-scrotal hernia. Additional causes to consider are scrotal tumors, idiopathic scrotal swelling, and Henoch-Schönlein purpura [2,10]. When evaluating the acute scrotum, features that suggest torsion of the epididymal cyst are the presence of a large cystic epididymal lesion with internal echoes and debris, which may represent hemorrhage or blood clots. A twisted pedicle may be seen on ultrasound but may not be detected at all times, since it may be very short, the adjacent inflamed tissues may obscure the pedicle, or severe tenderness may make adequate examination difficult.

Thus, the radiologist needs to keep in mind the diagnosis of epididymal cyst torsion in cases of acute scrotum where testis shows normal vascularity and other causes of acute scrotal pain are ruled out, and a complex epididymal cyst is present. Epididymal cyst torsion typically presents with a lack of blood flow on color Doppler ultrasound, distinguishing it from inflammatory conditions like epididymitis, which show increased blood flow due to inflammation. The role of color Doppler ultrasound is pivotal in this differentiation, as it provides vital information on blood flow patterns, essential for accurate diagnosis [11]. These insights ensure precise diagnosis and appropriate management in similar cases. Rarely, an epididymal cyst may be twisted but the patient may not have pain [1]. The necessity for surgery arises in cases of torsion or when ultrasound appearances are unclear. This case underscores the importance of prompt, accurate diagnosis and appropriate management, balancing the risks and benefits of surgical intervention versus conservative management.

Nevertheless, the final confirmation of epididymal cyst torsion is by surgical exploration. However, the radiologist can provide reassurance as to the normal vascularity of the testis and rule out other possible causes of acute scrotal pain. The key learning points from this case report are that in cases managed conservatively, patients and guardians should be educated about acute scrotum symptoms and when to seek immediate medical attention. Clinicians managing acute scrotal pain in patients should recognize the unique sonographic features of epididymal cyst torsion, understand the diagnostic capabilities of color Doppler ultrasound, and consider it as one of the differential diagnoses of acute scrotal pain.

## Conclusions

As epididymal cyst torsion is rare, healthcare professionals need to consider it as a differential diagnosis in cases presenting with acute scrotal pain. The case underlines the importance of prompt and accurate diagnosis to ensure effective treatment and avoid unnecessary medical procedures. Diagnostic challenges are notable, as epididymal cyst torsion can mimic more common conditions like testicular torsion. The report emphasizes the role of clinical examination and ultrasonography in differentiating these conditions. Lastly, it recommends that healthcare providers maintain a high index of suspicion in similar clinical scenarios and suggests guidelines for considering epididymal cyst torsion in future diagnoses, underscoring its impact on patient management and outcomes.
